# A stitch in time saves nine? A repeated cross-sectional case study on the implementation of the intersectoral community approach Youth At a Healthy Weight

**DOI:** 10.1186/s12889-015-2306-0

**Published:** 2015-10-08

**Authors:** Rianne MJJ van der Kleij, Mathilde R Crone, Theo GWM Paulussen, Vivan M van de Gaar, Ria Reis

**Affiliations:** Department of Public Health and Primary Care, Leiden University Medical Center, Postbus 9600 zone V-0-P 2300 RC, Leiden, The Netherlands; Academic Workplace (AWP) Public Health Zuid-Holland Noord, Leiden, The Netherlands; TNO Research Group Lifestyle, Leiden, The Netherlands; Department of Public Health, Erasmus University Medical Centre, Rotterdam, The Netherlands; Amsterdam Institute for Social Science Research, University of Amsterdam, Amsterdam, The Netherlands; The Children’s Institute, School of Child and Adolescent Health, University of Cape Town, Cape Town, South Africa

**Keywords:** Childhood obesity, Intersectoral community approach, Implementation, Qualitative methods, Process evaluation

## Abstract

**Background:**

The implementation of programs complex in design, such as the intersectoral community approach Youth At a Healthy Weight (JOGG), often deviates from their application as intended. There is limited knowledge of their implementation processes, making it difficult to formulate sound implementation strategies.

**Methods:**

For two years, we performed a repeated cross-sectional case study on the implementation of a JOGG fruit and water campaign targeting children age 0–12. Semi-structured observations, interviews, field notes and professionals’ logs entries were used to evaluate implementation process. Data was analyzed via a framework approach; within-case and cross-case displays were formulated and key determinants identified. Principles from Qualitative Comparative Analysis (QCA) were used to identify causal configurations of determinants per sector and implementation phase.

**Results:**

Implementation completeness differed, but was highest in the educational and health care sector, and higher for key than additional activities. Determinants and causal configurations of determinants were mostly sector- and implementation phase specific. High campaign ownership and possibilities for campaign adaptation were most frequently mentioned as facilitators. A lack of reinforcement strategies, low priority for campaign use and incompatibility of own goals with campaign goals were most often indicated as barriers.

**Discussion:**

We advise multiple ‘stitches in time’; tailoring implementation strategies to specific implementation phases and sectors using both the results from this study and a mutual adaptation strategy in which professionals are involved in the development of implementation strategies.

**Conclusion:**

The results of this study show that the implementation process of IACOs is complex and sustainable implementation is difficult to achieve. Moreover, this study reveals that the implementation process is influenced by predominantly sector and implementation phase specific (causal configurations of) determinants.

## Background

A worldwide increase in childhood obesity has been reported over the last decades [1–3]. In the Netherlands, an estimated 14 % of children have been classified as overweight or obese [1, 4]. Obesity often continues during adult life [5] and is linked to numerous adverse health outcomes [6–9]. As such, childhood obesity poses a major threat to public health [10], increases health care expenditures and as a consequence, constitutes an economic burden on society [11]. Intersectoral community Approaches to address Childhood Obesity (IACO) appear to have great potential to reduce and prevent childhood obesity [12–17]. An IACO aims to target the multiple determinants of childhood obesity by involving various stakeholders from within the community [15, 18, 19]. An example of a successful IACO that resulted in a decline of childhood obesity is the French ‘Ensemble Prévenons l’Obésité Des Enfants’ (EPODE) program. The conditions for effectiveness of EPODE are attributed to four center pillars; (a) political and organizational commitment, (b) collaboration between public and private organizations, (c) use of social marketing and (d) the support of scientific evaluation. As a result of its success, several EPODE-derived community approaches were developed [20–22]. In the Netherlands, the EPODE-derived JOGG approach (an acronym for Youth At a Healthy Weight, in Dutch) was installed [23, 24].

The innovation process of an IACO can be defined as the iterative cycle of program adoption, implementation and continuation [25]. This process is considered challenging; a translational gap between innovation development and implementation is often reported. Systematic insight into the delivery of innovation activities and the implementation of these activities by the intended user population is needed to develop strategies that have the potential to decrease this translational gap. Ultimately, these strategies can optimize the potential impact of the innovation [26–28].

Research on the implementation of interventions often focuses on fidelity: the extent to which an IACO is put into practice [29]. One critical aspect of fidelity is completeness, defined as ‘the proportion of IACO activities prescribed that is being put into practice’ [30]. Next to questions regarding completeness, research should also focus on the elucidation of determinants of completeness. Knowledge on these determinants is necessary to develop innovation strategies that have the potential for real change to occur [28, 31–35]. Only a dozen studies have specifically addressed the innovation process of IACOs [17, 36]. Even fewer studies have evaluated these processes longitudinally. Moreover, the quality of studies performed is not always up to par and determinants found to be critical still need to be (dis)confirmed by future research [37].

As part of a larger study [38], we therefore performed a repeated cross-sectional study on the innovation process of the JOGG “fruit- and water campaign”, evaluating both implementation and continued implementation of the campaign. This JOGG campaign took place in a disadvantaged neighborhood in a major city in the Netherlands, and aimed to promote healthy eating and drinking habits in children aged 0–12 years. Campaign strategies, mainly derived from social marketing, consisted of supplying promotional materials and organizing campaign activities such as educational supermarket visits and decorating water cans. Moreover, the campaign aimed issue a positive message to the target population (water and fruit are cool and hip!).

Our research questions were:

- To what extent were the JOGG fruit- and water campaign activities implemented as intended (completeness) from December 2011 to July 2014?
- What appeared to be the most critical determinants of the implementation of this campaign?
- Did determinants differ between the sectors involved (healthcare, educational, sports, welfare and private sector) *insert supporting information 1*, *sector categorization*)?
- Did determinants of implementation differ in time?

## Methods

### Design

This study was approved by the ethical committee of the Faculty of Psychology of the University of Leiden, reference number 8259652117. The evaluation was guided by the framework by Saunders et al. [27].

**Fig. 1:**
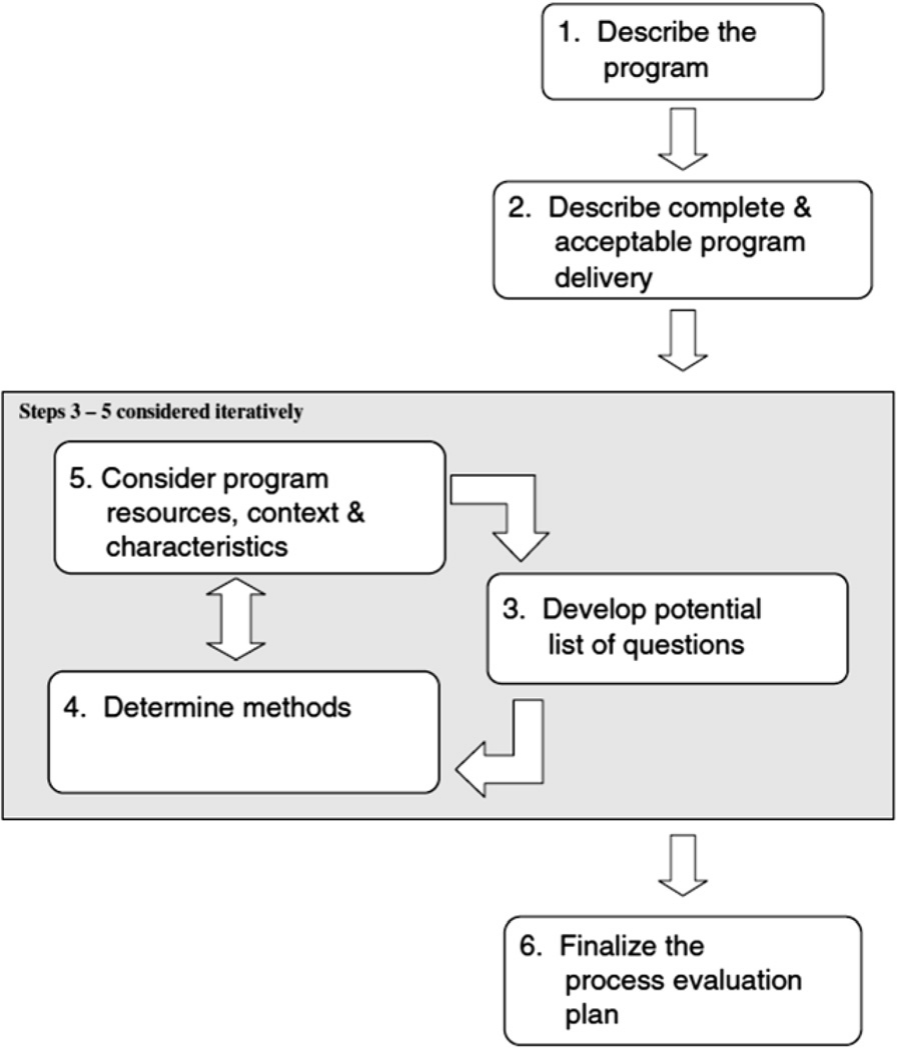
Overview of the framework by Saunders et al. [27]

**Fig. 2:**
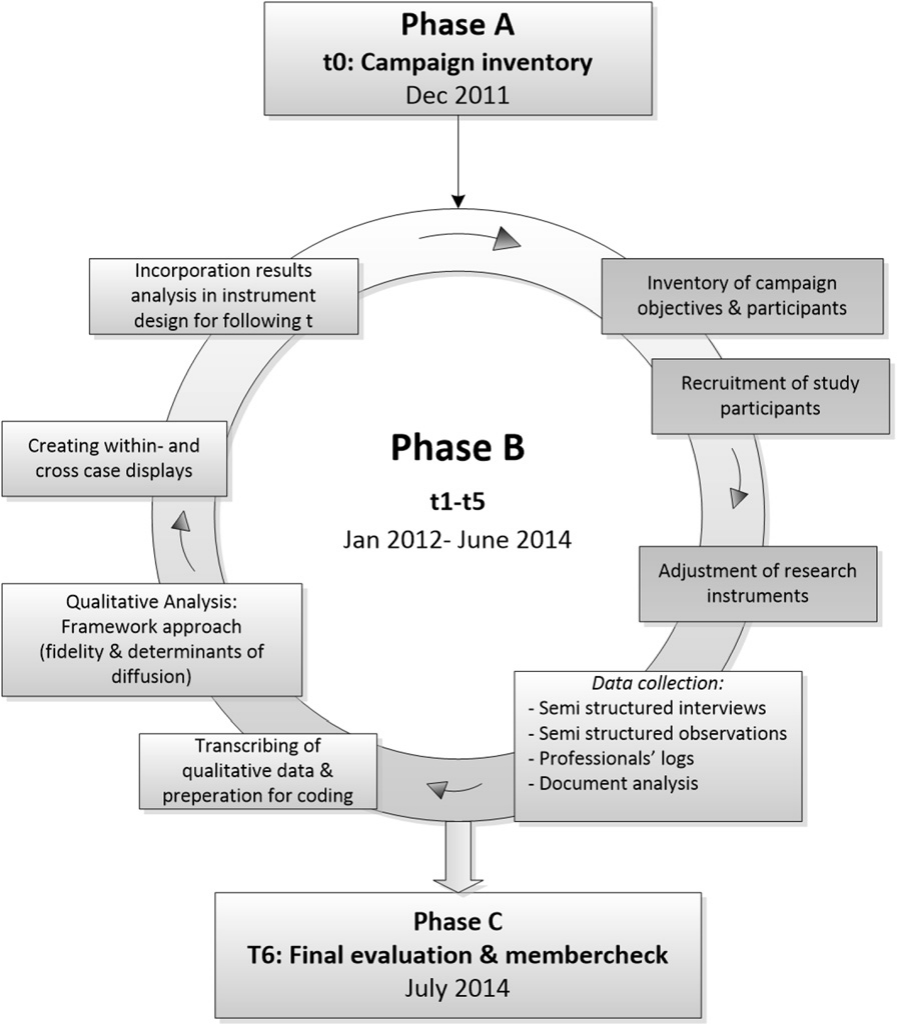
Overview of the research process

Research took place from the start of the campaign in December 2011 until its ending in July of 2014. As suggested by Saunders et al. [27] (Fig. 1), we first performed an inventory of the campaign’s setup (t0, research phase A) (Fig. 2). A blue print of the campaign design, setup and activities was then formulated. The implementation of the campaign was evaluated (research phase B, t1-t5) in five subsequent waves that coincided with ‘the booster months’ for either the water or fruit theme. For analytical purposes, we considered the first six months of campaigning as initial implementation, followed by mid-way implementation between 7–18 months, and continued implementation between 19–30 months. Thus, if an organization participated in the campaign from the start, initial implementation was assessed during t1, mid-way implementation during t2 and t3, and continued implementation during t4 and t5. A member check was obtained at t6 (research phase C).

### Sample & instruments

All research activities described in the next paragraph were performed iteratively during each research wave. Also, as stated by Saunders et al. [27], research instruments were adjusted before each wave following local developments and results of preliminary data analysis. Adjustments consisted, for example, of the addition of items to our interview topic list enquiring on ‘new’ determinants identified inductively via the preliminary data analysis.

At the start of each wave, all organizations meeting inclusion criteria were listed. Inclusion criteria consisted of (a) being situated within community boundaries, (b) receiving financial aid or materials from JOGG and/or (c) organizing activities within the context of JOGG. Via purposeful sampling [39], a selection of professionals working for the listed organizations was invited to participate in our study. Before participation, informed consent was obtained on audiotape from all participating professionals and transcribed verbatim.

To measure completeness (RQ1), prescribed campaign activities per organization were incorporated in observation checklists. If in vivo observation for certain activities was not possible, completeness was evaluated via the semi-structured interview detailed below. The checklist included items like “were fruit moments installed in your organization?” and “Did you organize the prescribed excursion to the local supermarket?”. Answers could be either yes (=1) or no (=0).

To evaluate determinants of implementation (RQ2), semi-structured interviews were conducted. The framework of Fleuren et al. [31] was applied as a lead for the selection of interview topics [40–42]. This framework distinguishes five major categories of determinants: (a) characteristics of the socio-political context, (b) characteristics of the organization, (c) characteristics of the intended user, (d) characteristics of the innovation and (e) characteristics of the innovation strategies. Interviews were held face-to-face and their duration varied from fifteen tot sixty minutes.

Document analysis was performed on planning documents, minutes of campaign team meetings and campaign manager’s log entries. Finally, field notes containing both notes from data collection and prejudgments of the researcher were taken into consideration.

Our study can be considered as ‘semi-action research’; we provided community stakeholders with study results after every wave and encouraged reflectivity. However, we did not advise them how to translate study results into improvements of the campaign. In this way, stakeholders were provided with the opportunity to optimize IACO implementation while keeping the level of data contamination to a minimum.

### Analysis

As for completeness (RQ1), all observations checklists were digitalized and transported to Microsoft Excel 2010. The proportion of all prescribed activities that were put into practice was then counted and a standardized score (percentage) per professional was calculated.

Interviews with professionals on the determinants of implementation (RQ2) were transcribed verbatim and transported to Atlas.ti for Windows version 6.2 (Scientific Software development, Berlin). They were then coded separately by two researchers (RK, SA), using a framework approach [43] derived from Fleuren et al. [31]. Data analysis was performed after each wave, and at t5 all previous analyses were re-evaluated. Next, data was further reduced by formulating within-cases and cross-cases [44]. Within-cases consisted of a narrative and a list of the most important facilitating and impeding determinants per professional. The subsequent cross-cases compared facilitating and impeding determinants per wave, sector and implementation phase. A determinant was classified as a ‘key determinant’ if it was indicated as a barrier or facilitator by more than 50 % of the professionals in the concerning cross-case.

### Causal configuration analysis

**Fig. 3:**
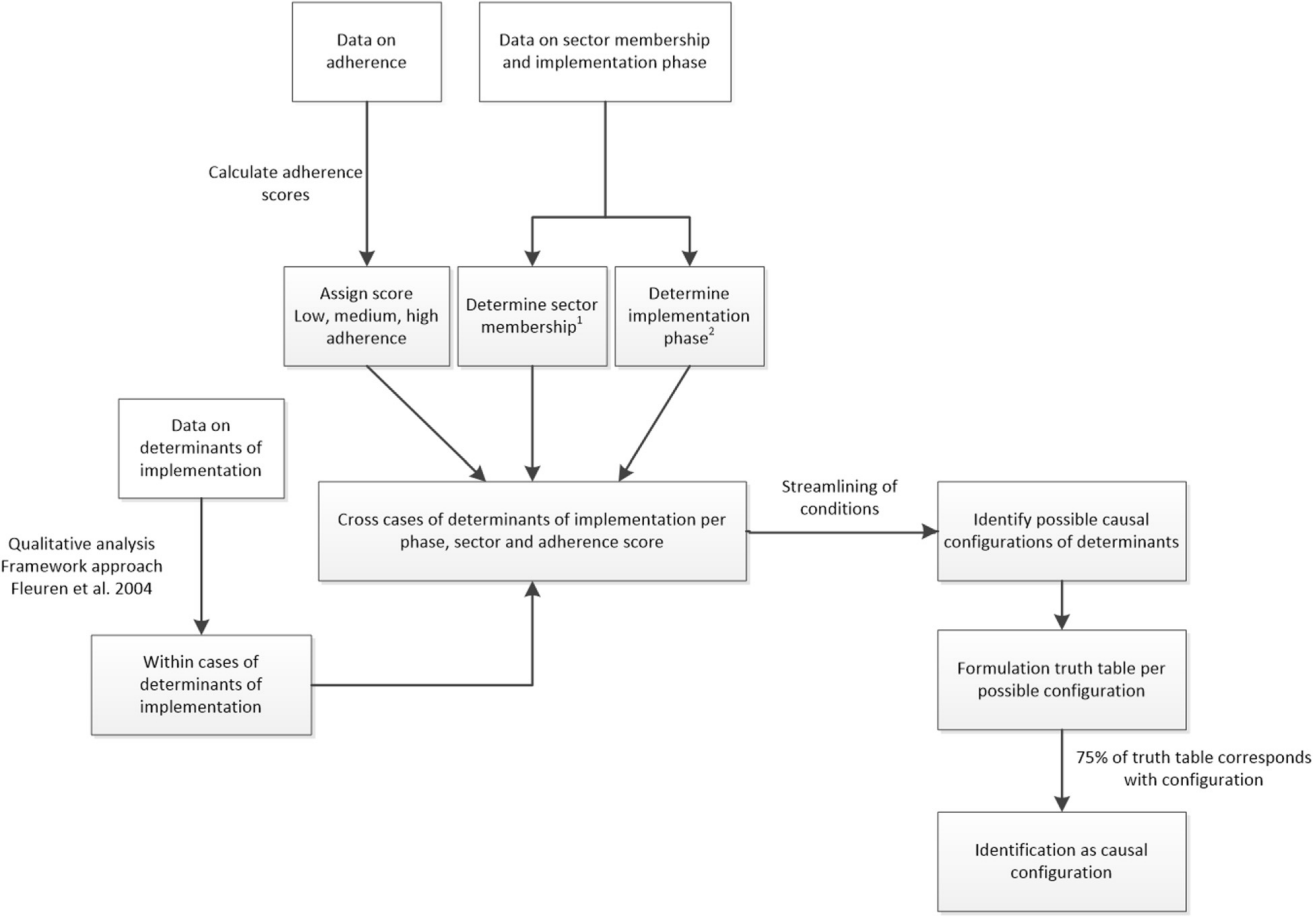
QCA derived causal configuration analysis

During cross-case analysis, we found that the determinants were not only self-contained, but seemed to be interrelated and occurring in causal configurations (e.g. presence of determinants A + B + C= > outcome X and presence of determinants B + C + D = > outcome Y). We considered using Qualitative Comparative Analysis (QCA) to analyze these configurations as QCA allows for interrelation analysis when different configurations generate the same outcome [45–48]. Moreover, this technique was successfully used to analyze similar configurations by Ordanini, Parasuraman and Rubura [47]. Our interviews however were semi-structured; participants did not provide information on exactly the same determinants. We therefore did not have data on the same determinants for all cases. To counter this challenge and at the same time preserve QCA assumptions, we translated QCA principles to a QCA derived causal configuration analysis (Fig. 3). We identified three outcome categories (low, medium and high completeness). Scores one standard deviation (SD) below the mean were categorized as low completeness, between one SD above and below the mean as medium completeness, and one SD above the mean as high completeness. We also determined sector membership and the implementation phase evaluated per professional. We then identified key determinants via cross-case comparison. After, we explored all possible causal configuration to see if the operator ‘or’ or ‘and’ between determinants could be placed (streamlining of conditions). Finally, truth tables were formulated for each possible configuration and a search for conforming and deviant cases was carried out. If contradictory cases were present, we decided that 75 % of professionals needed to confirm the configuration to be indicated as a causal configuration of determinants.

## Results

### Research phase A (t0): Campaign inventory & sample

The fruit and water campaign was part of the national JOGG approach and was financially supported by the local municipality. A campaign manager installed by the Municipal Health Services guided the installment of the campaign, in cooperation with a local campaign team. The campaign was setup as a manualized intervention. However, campaign activities were not prescribed via step-by-step instructions. Instead, activities were prescribed via less formalized instructions such as ‘install a water moment’ or ‘organize an excursion to the local supermarket’. Multiple organizations participated within the campaign, including schools, health organizations and private enterprises. All participating organizations were prescribed campaign activities directed towards the promotion of water and fruit consumption in children. However, the amount and specific content of prescribed activities varied per organization and sector, in accordance with their needs and possibilities. For example, supermarkets were not asked to organize any specific activities for children as they had indicated that insufficient time was available to facilitate such activities. Campaign activities could be divided into key- and additional activities for each sector (Table 1). Key activities were hypothesized by campaign management to be crucial for reaching the intended health promotion effect and were intended to be continued by the participating organizations after the end of the campaign. Additional activities were not intended to be continued after the campaign ended.

**Table 1:** Overview of key and additional campaign activities

Participating organization		Key activities	Additional activities
Educational	Primary schools	- Installment of fruit days & water moments - Teacher leads by example by eating fruit and drinking water - Informing parents about fruit & water consumption	- Distributing promotional materials (e.g. fruit baskets, water cans & crates, coupons for discounts at local enterprises, stickers, posters, banners, window foils, information cards for parents) -Engaging in campaign activities (e.g. the water song, excursions, watching television programs) -Promoting the campaign (displaying posters, banners and information cards) -Involving parents in campaign activities -Organize parent meetings in cooperation with the CJG
Health care	Centre for Youth & Family (CYF) Youth Health Care (YHC) Maternity/infant center	- Motivating target population to increase fruit & water consumption - Providing parents with advice on how to stimulate fruit and water consumption	- Distributing promotional materials -Promoting the campaign -Organize parent meetings in cooperation with schools
Social Welfare	Social Welfare organization Library	- Integration of campaign themes within regular activities - Professionals lead by example by eating fruit and drinking water	- Development of new activities related to campaign themes (e.g. decorating water carafes) -Engaging in campaign activities -Distributing promotional materials -Promoting the campaign
Sports	Sports organizations A& B	- Integration of campaign themes within regular activities - Professionals lead by example by eating fruit and drinking water	- Supplying fruit during sport events in the community -Integration of water theme in the ‘sports activity day’ for all schoolchildren. - Organizing sport activities for youth related to ‘water’
Private	Supermarket, household appliance stores	- Providing fruit free of charge to children - Providing discounts on fruit	- Promoting the campaign -Sponsoring of local activities -Selling campaign material

Our sample achieved reflects the complex and ever changing nature of the campaign; not all organizations could be included during all waves (Table 2). This was mostly due to organizations not being prescribed campaign activities or organizations declining (further) participation in the study. Limited time available for data collection by the researchers was also a reason for non-participation. During the study, priority was assigned to those organizations that were prescribed the most campaign activities and/or were considered as most critical for the impact of the campaign. To determine which organizations were most critical for impact, campaign management estimated the number of children that was going to be reached by activities, but also reviewed the content of the prescribed activities. For example, an organization providing a daily water moment during afternoon play activities to a hundred children was prioritized for study participation over an organization that performed a theatre play incorporating a water theme for twenty children on a one day event. Also, a larger number of interviews were conducted during t1 at schools B & C in comparison with school A. This difference was caused by the fact that researchers from an aligned college were conducting an evaluation of fruit- and water consumption at schools B&C at the time of our study. As they were also qualified to perform qualitative research and were already visiting these schools, they were asked to interview the teachers enquiring on implementation using our interview topic list next to their own study. Hence, additional interviews were thus acquired during t1 at schools B&C.

**Table 2:** Sample achieved

	T1		T2		T3		T4		T5	
	Int	Obs	Int	Obs	Int	Obs	Int	Obs	Int	Obs
Educational										
Primary school A	3	2	2	2	2	3	2	2	PD	
Primary school B	7	7	3	2	2	3	2	2	2	2
Primary school C	9	9	2	2	2	3	2	2	PD	
Primary school D	PD		1	1	2	3	2	2	2	2
Health care										
Centre for Youth & Family	1	1	1	1	-	1	1	1	1	1
Youth health services	2	1	3	3	1	1	2	2	1	1
Social welfare and sport										
General welfare organization	1	1	1	1	2	2	1	1	1	1
Library	NP		-	1	Closed due to budget curtailments					
Sports organization A	NP		NP		1	1	0	1	NP	
Sports organization B	NP		NP		1	1	0	1	NP	
Private										
Supermarket A	NP	1	1	1	1	1	1	1	1	1
Supermarket B	NP	1	1	1	1	1	1	1	1	1
Household appliance storeA	NP		1	1	0	1	NP		NP	
Household appliance store B	NP		1	1	0	1	NP		NP	
Household appliance store C	NP		1	1	0	1	NP		NP	

*Int* number of interviews, *Obs* number of observations, *NP* not prescribed campaign activities, *PD* declined (further) participation in study

### Research phase B: Evaluation of implementation (t1-t5)

#### General findings

Completeness ranged from 0-100 % throughout sectors and implementation phases (Table 3). Overall, completeness of key activities was higher than for the additional activities The highest levels of completeness were observed in both the educational and health care sector. A majority of organizations showed a notable decline in completeness from t2 to t3.

**Table 3:** Completeness of key- and additional activities

	T1		T2		T3		T4		T5	
	% Key	% Add	% Key	% Add	% Key	% Add	% Key	% Add	% Key	% Add
Educational										
Primary school A	75	29	54	27	17	4	75	32		
Primary school B	33	24	83	39	100	33	100	64	50	100
Primary school C	63	39	88	39	50	35	42	37		
Primary school D					50	33	75	36	100	100
Health care										
Centre for Youth & Family	100	25	100	75		75	100	80		100
Youth health services	83	43	100	71	67	67	75	75	100	100
Social welfare and sport										
General welfare organization	67	50	0	14	67	17	25	100	100	100
Library			50	50						
Sports organization A					0	0	0	0		
Sports organization B					33	0	0	0		
Private										
Supermarket A			100			100	100	100	100	0
Supermarket B			0			0	100	80	33	0
Household appliance storeA			100	33	0	0				
Household appliance storeB			100	0	0	0				
Household appliance store C			100	0	0	0				

*Key* Key activities, *Add* additional activities

Twenty-four key determinants were identified; ten facilitators and fourteen barriers (Table 4). Overall, high ownership towards campaign goals (feeling psychologically tied or attached to campaign goals [49]) and high compatibility of the campaign with existing working procedures were most cited as facilitators to implementation. Most frequently named barriers were a lack of reinforcement strategies for ongoing use of the campaign (e.g. a training or new promotional materials), a low priority for campaign use, low procedural clarity and incompleteness of campaign materials (e.g. insufficient quantity of campaign materials, campaign lacking classroom teaching materials). Eleven causal configurations were identified across four sectors (Table 5); ten configurations were related to a medium to high level of completeness. For the healthcare as well as the educational sector, we identified a causal configuration related to both medium and high completeness for an identical implementation phase. Across these sectors, the facilitators identified in the medium and high completeness configuration were mostly similar, whereas barriers were halved or not present at all in the high completeness configurations. For the private sector, a low and high completeness configuration was identified for continuing implementation. Barriers identified were identical for both low and high completeness, whereas facilitators were only absent in the low completeness configuration. Details per sector on levels of completeness, determinants and configurations are described below.

**Table 4:** Key barriers & facilitators per sector per implementation phase

	Educational			Health care			Private			Welfare & sports		
	Int	Mid	Cont	Int^a^	Mid	Con	Int	Mid	Cont	Int	Mid	Cont
Key facilitators												
Campaign compatible with existing work procedures	●	●			●	●				●		
Possibility to adapt campaign to local needs	●	●					●					●
↑ ownership for campaign use		●	●		●	●	●	●	●	●		
↑ self-efficacy for campaign use			●								●	
Uptake of campaign use in daily working routine			●									
*Availability of internal campaign coordinator*					●							
↑ support from campaign manager					●					●		
Regular evaluation of campaign implementation					●							
Campaign use cause advantages											●	
*Compatibility of campaign goals and goals of organization*												●
Key barriers												
↓ procedural clarity	●						●	●				
Campaign use causes disadvantages	●											
↓ priority assigned to campaign use		●	●		●							
↓ durability of campaign materials		●										
*Lack of campaign reinforcement strategies*			●						●			●
Campaign is considered incomplete					●	●		●				
*Chaotic organization of campaign*						●				●		
*Incompatibility of campaign goals and goals of organization*							●					
↓ participation of target population in campaign								●	●			
High turn-over of staff								●				
*Lack of experiencing a shared commitment for campaign use with community partners*									●			

*Int* Initial implementation, *Mid* mid-way implementation; *Con* continued implementation; ^a^too little data available to draw conclusions; Determinants outside of the scope of the Fleuren framework are italicized

**Table 5:** Causal configurations of determinants

Sector	Phase	Outcome (completeness)	Causal configurations^a^	# cases
Educational	Initial implementation	Medium	*Campaign perceived as disadvantageous* AND *↓ procedural clarity* AND (**↑ possibility to adapt campaign to local needs** OR **↑ ownership for campaign use** OR **campaign compatible with existing work procedures**)	7 out of 10 cases
	Initial implementation	High	*No barriers named* AND **Possibility to adapt campaign to local needs** AND **↑ ownership of campaign use**.	2 out of 2 cases
	Mid-way implementation	Medium / High	(*↓ priority assigned to campaign use* OR *↓ durability of campaign materials*) AND **Possibility to adapt campaign to local needs** AND (**↑ ownership for campaign** OR **campaign compatible with existing work procedures**)	9 out of 11 cases
	Continued implementation	Medium	(*A lack of reinforcement strategies* OR *campaign use not included in task orientation*) AND (**Possibility to adapt campaign to local needs** OR **↑ ownership of campaign use** OR **↑ self-efficacy**)	4 out of 4 cases
	Continued implementation	High	*No barriers named* AND **Uptake of campaign use in daily working routine** AND (**↑ ownership of campaign use** OR **↑ self-efficacy**)	5 out of 6 cases
Health Care	Continued implementation	Medium	*Campaign perceived as incomplete* AND *Chaotic organization of campaign* AND **campaign compatible with existing work procedures**	2 out of 2 cases
	Continued implementation	High	*A lack of reinforcement strategies* AND **campaign compatible with existing work procedures** AND **↑ ownership of campaign use**	2 out of 2 cases
Private	Continued implementation	Low	*↓ participation of target population in campaign* AND *Lack of feeling part of collaboration in community*	2 out of 2 cases
	Continued implementation	High	*↓ participation of target population in campaign* AND *Lack of feeling part of collaboration in community* AND (**perceiving campaign use as personal duty or obligation**)	2 out of 2 cases
Welfare	Initial implementation	Medium / High	(*↓ procedural clarity OR campaign perceived as incomplete*) AND (**campaign compatible with existing work procedures** OR **↑ ownership of campaign use**)	3 out of 3 cases
	Continued implementation	Medium / High	*A lack of reinforcement strategies* AND **possibility to adapt campaign to local needs** AND **Uptake of campaign use in daily working routine**	2 out of 2 cases

^a^Italic = barrier, Bold = facilitator

#### Implementation per sector

##### Educational sector

Completeness of key activities in schools varied between 33-75 % during initial implementation. Overall, low completeness in schools during initial implementation was associated with a lack of procedural clarity or unforeseen negative experiences during implementation (for example chaos caused by preschoolers having difficulties making it to the bathroom).

We need to plan extra toilet breaks… look, he (student) just peed in his pants and that is just because he drank a lot of water due to the water campaign. *(Teacher school B)*

Throughout mid-way implementation, completeness declined to 50 % or less for schools A & C. Teachers from these schools ascribed this decline to the hectic working schedule they followed, which made prioritizing the promotion of a healthy lifestyle difficult.

We have been so busy the last couple of years, at a certain moment you think ‘I don’t even know the name of this student in my class’. So I think.. Yes, our main priorities lie elsewhere, not with the water campaign. *(Teacher school A)*

In schools B & D, completeness stayed above 50 % during mid-way implementation. This was often attributed to the program’s compatibility with pre-existing practices (such as the school schedule) or to the possibility to adapt non-essential elements of the campaign (such as timing of water moments) to their own needs.

During continued implementation, completeness recovered from 17 to 75 % at school A and remained above 50 % for schools B&D. Recovery of the completeness rate for school A was attributed to the instalment of an coordinator who advised on how to integrate campaign activities in daily routines (such as combining a play-time break with a water moment). Overall, high levels of completeness in continued implementation were associated with high levels of self-efficacy (beliefs about the ability to reach campaign goals).

At school C, completeness stayed below 50 % during continued implementation. Teachers from school C often attributed their low level of completeness to the lack of reinforcement strategies available for campaign use, such as the provision of a training or new promotional materials.

At first, everything was new, they (students) all had their campaign water bottles on their desks and it was very hip and happening! But, yeah, I don’t know, it is just not cool anymore now. *(Teacher school C)*

As for causal configurations, during both mid-way implementation and continued implementation professionals displaying high completeness indicated the same facilitators as professionals displaying medium completeness. However, professionals displaying high completeness indicated no key barriers (Table 5).

##### Health care sector

Within the health care sector, completeness of key activities varied from 67–100 %. Professionals stated that compatibility of the campaign with their daily practices facilitated implementation.

It (the campaign) is now part of my job. So I automatically integrate it into my daily work procedures, this makes the execution easier. *(CYF*, *nurse)*

Also, the presence of an internal coordinator to assist campaign implementation was named as a facilitator. During continued implementation, incompleteness of campaign materials was named as a key barrier by professionals. For instance, the distribution of campaign materials was often hindered which resulted in too little campaign materials being available.

With regard to configurations, professionals showing medium completeness during continued implementation stated campaign materials were incomplete and the campaign was poorly organized but found that the campaign to be compatible with existing procedures. All professionals displaying high levels of completeness mentioned the campaign to be compatible with existing practices and stated that they felt high ownership towards achieving the campaign goals. However, they cited the campaign lacked reinforcement strategies.

##### Welfare & sports sector

For the general welfare organization, a significant decline in completeness of key activities was observed from initial to mid-way implementation. After an initial uplift in completeness (67 %), levels declined again to 25 % at the beginning of continued implementation but reached a 100 % at the end of this phase. Recovery of completeness was mostly attributed to the adaptation of non-essential campaign components to local circumstances and the subsequent uptake of these activities in daily routine.

It costs quite a lot of time to organize a water or fruit booster. But because we now implement it (campaign activities) during our regular activities, it is working out fine! … we for example organized a community walk yesterday, and we provided children with a healthy snack. So it (campaign objectives) just became a standard procedure. *(Social Welfare Organization*, *Social worker children)*

Causal configurations revealed that professionals displaying medium to high completeness during implementation all reported that the campaign was incomplete or campaign procedures were unclear, but that they felt highly committed towards the goals to be achieved or found the campaign was compatible with existing work procedures.

Sports organizations showed low completeness (0-33 %) during initial implementation, and ceased campaign activities after this phase. This was mostly attributed to the incompatibility of the campaign with existing working procedures and incompleteness of campaign materials. They reported a mismatch between the equipment needed on the sports field (water tanks) and equipment received (water cans). Moreover, they reported that the number of pupils did not equal the promotional materials received and the promotional materials was delivered while the organizations were closed for the winter break.

##### Private sector

The household appliance stores opted out of the campaign after initial implementation. One supermarket showed a completeness score of 100 % during continued implementation, the other supermarket displayed lower levels of completeness (33 %). Ownership of campaign goals was cited as a key facilitator in all implementation phases. During initial implementation, the incompatibility of the campaign goals with the goals of the organization was identified as a key barrier.

I didn’t understand the campaign method, I thought the mega fruit cup was a hideous thing, that ruined the image of my shop!. … we have to draw a line somewhere, we are a supermarket and not the extension of municipal programs. *(Supermarket B*, *manager)*

Not having a feeling of shared commitment with community partners to implement the campaign was cited as a key barrier throughout continued implementation.

As i have experienced it, the campaign is very standalone instead of coming together with multiple partners and discussing ‘what are we going to do about it’? I think this would open a window of opportunities. *(Supermarket A*, *manager)*

Causal configurations revealed that professionals displaying low completeness during continued implementation stated the participation of the target population was lacking and that they did not experience a shared commitment for campaign use with community partners. Professionals displaying high completeness in this phase also expressed these barriers but stated they perceived campaign use as a personal obligation.

## Discussion

The aim of this study was to evaluate completeness of the activities prescribed for the JOGG fruit- and water campaign and to identify the most critical implementation determinants.

Overall, completeness of activities was highest for the general welfare organization, and the educational and healthcare sector organizations. Moreover, completeness was higher for key activities than for additional activities. A decline in completeness was observed for a majority of sports- and private sector organizations after (initial) implementation, and a general decline in completeness was half way the study period. Key barriers identified varied more than key facilitators. High ownership for campaign goals and high compatibility of the campaign with existing procedures were most often cited as facilitators, whereas a lack of reinforcement strategies, a low priority for campaign use, low procedural clarity and incompleteness of campaign materials were most frequently indicated as a barrier. Eleven causal configurations of determinants were identified across sectors and a majority of configurations was related to medium or high levels of completeness.

### Implications of findings

Previous research corroborates our findings that levels of completeness differs greatly between sectors and implementation phases [50–52] and that sustainability of IACOs is hard to accomplish [53]. The general decline in completeness observed halfway the study period (t3) could be explained by the temporary incapacitation of the campaign manager, in combination with the set-up of the IACO. The water- and fruit campaign was highly manualized and delivered top-down, which has been associated with lower levels of ownership [54]. Hence, we argue that in particular in such a top-down implementation approach, the lack of campaign managers’ support in combination with this lower levels of ownership could explain the poor IACO sustainability [55]. Lack of the support of campaign management or lack of ownership were however not explicitly reported as barriers by the professionals; they solely reported a less orderly campaign organization and incomplete delivery of the campaign materials at t3.

The framework of Fleuren et al. [32] proved partly inadequate to identify determinants of implementation of IACOs; seven key determinants identified fell out of the scope of this framework. These determinants, such as ‘difficulty to collaborate with community partners’ seem to be more specific to the intersectoral, community-based characteristics of IACOs, and are in line with other studies on the implementation of IACOs [36, 50–53, 56–67]. Determinants identified were, to a great extent, sector and implementation phase specific. For example, perceiving campaign implementation as a personal duty or obligation was identified only as a facilitating determinant for the private sector, whereas uptake of the campaign in daily working routine was only named a facilitator for the educational sector. We therefore argue that implementation plans and strategies should be tailored to sector and implementation phase specific determinants. In addition, adjustments to implementation plans and strategies should be verified and discussed with professionals throughout the implementation process to ensure an optimal fit with the implementation context. This course of action responds to the need expressed by professionals from four out of five sectors to adjust the campaign and its strategies to local needs. This so called ‘mutual adaptation approach’ provides an opportunity to obtain site-specific feedback from local professionals, and was named in previous studies as a facilitator for institutionalization of health promotion programs [68] and the implementation of complex innovations in cancer care [69].

An interesting distribution of barriers and facilitators was found among the causal configurations identified for the educational and private sector. In the educational sector, the medium and high completeness configurations identified contained mostly identical facilitators. Most facilitators named in these configurations were internal, such ‘self-efficacy’, ‘ownership’ and ‘task orientation’. The distribution of barriers however differed between these configurations; the medium configuration contained mostly external barriers (such as procedural clarity of the campaign), whereas the high configuration contained no barriers at all. This could imply that, although the same facilitators were present, the absence of certain external barriers could be decisive to achieve implementation success in this sector. For the private sector, barriers identified for both the low and high configurations were similar and mostly external, namely ‘low participation of the target population’ and ‘not feeling part of collaboration in community’. However, an internal facilitator was only present in the high configuration, namely ‘perceiving campaign use as personal duty or obligation’. This could imply that, independent of the external barriers present, perceiving the campaign as a personal duty or obligation is a decisive factor for implementation success in the private sector. However, the fact that the barriers named in these configurations were mostly external and the facilitators named were mostly internal could indicate some form of self-serving bias [70] is present in our data. Hence, participants were perhaps inadvertently more prone to erroneously attribute success to internal factors, and failure to external factors.

The casual configurations extracted from our data indicate that a set of determinants can jointly lead to implementation success or failure. We therefore argue ‘the whole to be greater than the sum of the parts’ and that implementation might benefit more from implementation strategies based on all the configuration determinants combined, than of strategies based on single determinants. Further research testing the effect of such implementation strategies integrating causal configurations in its entirety is warranted to investigate this assumption. Finally, it should be noted that the analysis of causal configurations in qualitative research is still in its infancy [71]. Although, in our opinion, the of use an adapted version of QCA was the best choice to systematically analyze these configurations at this moment in time, readers should keep in mind that no golden standard yet exists and only a limited number of cases were studied. Hence, the reported results should be interpreted with caution.

### Strengths & weaknesses

To ensure a systematic, theory-based study design, the framework of Saunders et al. [27] was used to guide our process evaluation. This framework allowed for a sharpened focus in data collection as well as the iterative adjustment of research methods in accordance with (preliminary) results. Although (preliminary) study results were used to adjust research methods, we did not use these results to adjust or improve campaign plans and strategies. Instead, the interaction with practice was guided by a semi-action research design. Hence, we presented the study results to stakeholders after every wave, but did not recommend any changes or alterations to campaign implementation. We chose this approach as to enhance stakeholders’ ability to optimize IACO implementation whilst ensuring a minimal level of data contamination. However, although we anticipated that the mere provision of results would encourage practice to optimize implementation plans, due to time limitations and lack of expertise little could be done by campaign management and practioners with the study results provided. We argue that Participatory Action Research (PAR) [72], in which researchers aid practioners with the translation of research findings into implementation strategies, could perhaps enable practice to take optimal advantage of process evaluation data. A review by Cook [73] revealed that PAR led to the translation of research finding into community action in fourteen out of the twenty studies reviewed. The benefits of PAR would therefore, in our opinion, outweigh the possibility of data contamination, which is perhaps partly inevitable when performing IACO process evaluations.

Several other methods were employed to optimize the credibility, objectivity and internal validity of our data [44, 74]. We collected data via in vivo observation, in contrast with most implementation studies who merely rely on self-reports [30]. Furthermore, data was recorded and transcribed verbatim, analytic software and a framework approach were used for data analysis and further data reduction was performed using theoretically approved methods [44]. Also, coding was performed by two researchers and the principal researcher (RK) kept a log about her opinions and prejudgments to increase awareness en reflexivity, reducing moderator bias [75].

One limitation of this study is the selection of participants. Due to the complex and rapidly evolving nature of the campaign investigated, selection of participants was not at random but per opportunity. This makes selection bias possible [76]. Also, we could not evaluate the implementation process of the same individual(s) for every organization at every measurement. This was partly due to ‘research fatigue’ [77]; for example schools stated they already participated in a number of research activities and therefore wanted to spread the ‘burden’ of study participation by alternating study participation among teachers across measurements. But also the complex and dynamic character of community state of affairs influenced participation; for instance supermarkets showed a high turnover of staff which made it impossible to include the same individual throughout measurements. We countered these sampling issues by ensuring that if the persons included were not similar across measurements, the function or role that the included professionals fulfilled per organization was similar. For example, at schools we always included a teacher from elementary- and middle school, and for supermarkets we always included the floor manager.

## Conclusion

This study underlines the complexity of process evaluation of IACOs; the research environment is ever changing and research plans need to be constantly adapted following local developments. Moreover, a participatory action research approach should be considered to enable the swift implementation of study results into practice. Results of this study provide some leads for the formulation of implementation strategies and plans, but more research is needed to (dis)confirm these findings and their generalizability. Tailoring of implementation plans and strategies should be based on a combination of the determinants identified in this study within the context of a mutual adaptation strategy. Hence, ‘stitches in time’ are needed to allow professionals to complement and verify the tailored strategies developed throughout the implementation process.

**Table:** 

**Lessons learned**
• Research plans need to be adapted iteratively to local developments;
• The translation of research findings into practice could possibly be optimized by the use of participatory action research (PAR);
• A complete, understandable IACO that is compatible with and considered relevant by practice can facilitate IACO implementation
• As some determinants appeared in configuration per sector and phase, implementation might benefit from consideration of these determinants in unity, rather than considering single determinants;
• Implementation plans and strategies should be tailored to sector- and implementation phase specific (combinations of) determinants, and should be based on a mutual adaptation strategy (“stitches in time”).
